# Public health system readiness to treat malaria in Odisha State of India

**DOI:** 10.1186/1475-2875-12-351

**Published:** 2013-10-02

**Authors:** Mohammad A Hussain, Lalit Dandona, David Schellenberg

**Affiliations:** 1 Indian Institute of Public Health, Bhubaneswar, Odisha, India; 2 Public Health Foundation of India, New Delhi, India; 3 Institute for Health Metrics and Evaluation, University of Washington, Seattle, WA, USA; 4 London School of Hygiene and Tropical Medicine, London, UK

**Keywords:** Health workers’ readiness, Health system readiness, Malaria, Drug policy, Implementation, India

## Abstract

**Background:**

Early diagnosis and prompt treatment is a cornerstone of malaria control. In India, artemisinin combination therapy (ACT) became the first-line treatment for falciparum malaria and rapid diagnostic test (RDTs) kits were recommended for use at the grass-root level in the new malaria treatment policy (2010). Odisha State contributes about one-fourth of the total Indian malaria burden and 40% of falciparum infection. The present study assessed the health system readiness to deploy RDTs and ACT for malaria control across the State.

**Methods:**

Data collection was carried out from February to July 2012. Five of Odisha’s 30 districts were selected through stratified random sampling, with stratification based on the phased roll-out of ACT and RDT. Two administrative 'blocks’ were selected randomly in each district and data collected through health facility, auxiliary nurse midwives (ANMs) and accredited social health activist (ASHAs) assessments. Key informant interviews were conducted with individuals involved in the implementation of the malaria control programme.

**Results:**

Of the 220 ANMs interviewed, 51.4% had been trained in malaria case management, including the use of ACT and RDT. A high proportion of ANM (80%) and AHSA (77%) had the necessary level of knowledge to be able to use RDT for malaria diagnosis. The proportion of ASHAs trained on malaria case management was 88.9% (209/235). However, 71% of ANM and 55% of ASHAs usually referred falciparum-positive patients to the health facility for treatment, the major reason for referral being the non-availability of drugs at the ANM and ASHA level.

**Conclusion:**

The relatively high level of knowledge about how to diagnose and treat malaria at the grass-root level was undermined by the poor availability of RDTs, ACT and primaquine tablets. This was associated with an unnecessarily high referral rate and potential delays in the treatment of this potentially life-threatening infection. Improvements in the supply chain for RDTs and ACT could dramatically enhance the effectiveness of malaria control in Odisha.

## Background

Malaria remains a major public health challenge in India, contributing two-thirds of the parasitologically confirmed malaria cases in the Southeast Asia region [1]. Disease transmission is geographically limited, however, with Odisha, Jharkhand, Madhya Pradesh, Chhattisgarh and the north-eastern states contributing the bulk of morbidity and mortality in the country [2-4].

The National Drug Policy on Malaria, first formulated in 1982, has been revised periodically to counter the effect of drug resistance [5]. In 2010, artemisinin combination therapy (ACT) became the first-line treatment of *Plasmodium falciparum* malaria. ACT was recommended for use at the community level by auxiliary nurse midwives (ANMs), accredited social health activists (ASHAs) and other community health volunteers [6]. It was also recommended that rapid diagnostic test (RDTs) kits should be introduced to these cadres where microscopy was not available. Successful implementation of the policy therefore requires the availability of appropriately trained health personnel, RDTs and ACT at the point of care [7,8].

Far more effort has gone into determining which anti-malarials are efficacious than determining how to deliver them effectively [9]. Several studies have drawn attention to the insufficiency of the formal public health sector to guarantee the availability of drugs, training and guidelines several years after the introduction of new drugs [10-12]. These studies serve as a reminder that having recommendations for efficacious drugs do not necessarily translate into health system preparedness to deliver the new medicines to target groups.

This paper reports a study from the State of Odisha, on the eastern coast of India, which has a population of 42 million, 3% of the total Indian population [13], but carries over 25% of the national malaria burden, including 42% of *P. falciparum* infections [14]. Malaria remains an important cause of morbidity and mortality in the State, with some estimates in excess of 50,000 deaths per year [2]. Through health facility surveys and interviews with community workers, the study attempted to evaluate the system readiness to deploy RDTs and ACT for malaria control across the State.

## Methods

### Study setting

Odisha State has extensive remote and underserved areas, with about 40% of the population living below the poverty line. Odisha is divided into 30 districts, each divided into administrative blocks with an average population of 120,000 to 150,000. The state public health system has a three-tier structure with a district hospital providing secondary care at the district headquarters. At the block level there is a community health centre (CHC) providing preventive and curative health services. Under each CHC, three to five primary health centres (PHCs) provide primary health care services to a population of about 25,000 to 30,000 each. Under each PHC there are four to six subcentres, each managed by an ANM and serving a population of around 3,000 to 8,000. Each subcentre covers five to six villages, each with a community health worker, known as an accredited social health activist (ASHA), who is supervised by an ANM and provides care for about 1,000 people. ASHAs are local women trained to act as health educators and promoters in their communities and receive a performance based incentives for their activities.

Considering the operational feasibility and optimal use of allocated resources, the National Vector Borne Disease Control Programme (NVBDCP) state office has oriented the district authorities to distribute drugs and RDT kits as follows: endemic areas with an annual parasite incidence (API) > five, RDTs, ACT and other oral anti-malarials should be available at village level for ASHAs to use. In areas (moderately endemic) with an API > two but < five, RDTs and anti-malarials, including ACT, should be made available to the ANM, subcentre level and above, but not at the village level. In areas where the API < two or where malaria is non-endemic, the ANM should have RDT, chloroquine (CQ) and primaquine (PQ). Every febrile patient with possible malaria should be diagnosed with quality microscopy or RDT and, if found positive, should be treated with a full course of ACT (artesunate three days + sulphadoxine-pyrimethamine one day) and PQ for *P*. *falciparum,* and with CQ (for three days) and PQ (14 days) for *Plasmodium vivax* cases. The NVBDCP state office is responsible for preparing and circulating to all cadres of personnel the malaria treatment guidelines and drug dosage charts. Five colour-coded ACT packs are supplied for different age groups (under-one year, one to four years, five to eight years, nine to 14 years, and 15 or more years or adult pack).

Three years’ API data (2009-2011), obtained as routine data by the State, for the blocks included in the survey were reviewed. The stability of API over time was assessed and the implemented malaria diagnosis and treatment strategies were compared with the State recommended strategy.

### Survey design

This cross-sectional study was carried out between February and July 2012 and included a government public health care facility assessment and key informant interviews of personnel involved in implementation of malaria control programme in the State.

### Sampling frame and sampling technique

**Figure 1:**
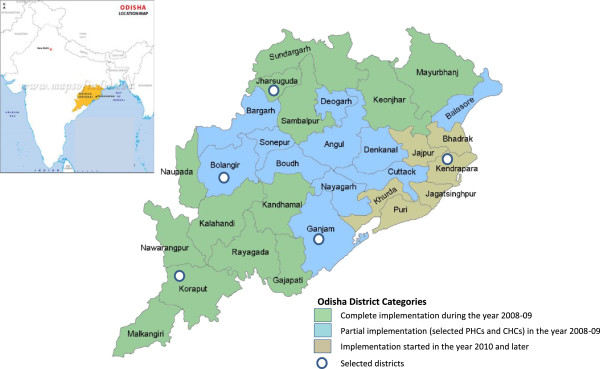
Map of Odisha showing selected districts and artemisinin combination therapy/rapid diagnostic testing policy implementation dates.

**Figure 2:**
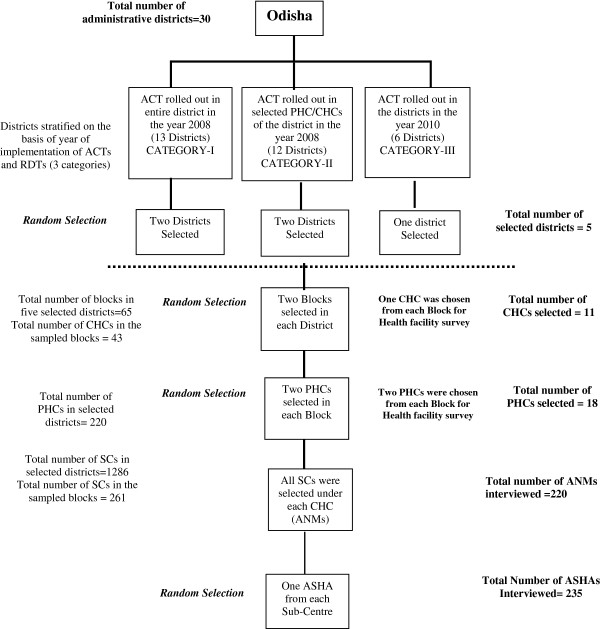
Sampling framework for selection of health facility and study participants.

Health facilitates and study participants were selected through multistage stratified sampling based on the intended timing of roll-out of ACT and RDTs. A 2008 drug policy revision recommended ACT roll-out in 13 highly endemic districts (Category-I) plus 39 PHCs from another 11 moderately endemic districts (Category-II). These areas were targeted by the policy because of the level of CQ resistance, *P. falciparum* disease burden and transmission. The third category of districts (Category-III) was malaria non-endemic where ACT was supposed to be implemented as per the policy revision of 2010. Five districts (Figure 1) were selected using stratified random sampling, two randomly from the first two categories and one randomly from the third category. Two administrative blocks were then randomly sampled within each sampled district (Figure 2).

### Study participant selection

Health workers providing diagnostic and treatment services at different levels of the health system were selected for interview. All ANMs in selected blocks were included in the study, with the intention to interview about 50 ANMs from the two blocks selected in each district. If the number of ANMs in the two selected blocks in the district was less than 50, then an additional block was selected randomly and ANMs were selected randomly from the newly selected block until the sample size was reached. The list of ASHAs was prepared in discussion with the ANM and then one ASHA was selected at random for each subcentre for interview at her residence. If, in spite of two visits, the ASHA could not be contacted then another ASHA was selected from the same subcentre. Those ANMs and ASHAs who could not be contacted in their respective villages were interviewed during the weekly meeting at their respective PHCs or monthly meeting at CHCs. Simultaneously, in order to have an in-depth understanding about the malaria diagnosis and treatment practice prevalent in their area, a subsample of one in seven ANMs and ASHAs was systematically selected and interviewed in detail.

All data were collected by two specially trained field investigators. The fieldwork was supervised by the lead author. For quality assurance during data collection, 5% of the data forms completed in each block by field investigators were randomly checked, and 50% of all ANMs and ASHAs in-depth interviews were conducted in the presence of the lead author. All interviews with key stakeholders at district and state level were conducted by the lead researcher himself.

### Tools and techniques for data collection

Three different strategies were used for data collection. First, all selected ANMs and ASHAs were interviewed using a semi-structured questionnaire. Secondly, health facility assessments were conducted for selected CHCs and PHCs. Thirdly, in-depth interviews were held with key stakeholders in malaria control at district and state levels. Separate semi-structured interview schedules were developed, pilot-tested and translated into the local language, Odiya, for collection of information from the ANMs and ASHAs. This assessed their knowledge of malaria and new treatment guidelines, exposure to in-service training, the availability of RDTs for malaria diagnosis and ACT for treatment. The duration of stock-outs of anti-malarials and RDTs, if any, during the preceding three months was assessed. In addition, the availability of treatment guidelines and job aids for malaria case-management were also explored. Health facility questionnaires explored the different kinds of malaria diagnosis and treatment services provided at the facility, the availability of ACT and RDTs on the day of the survey, evidence of any stock-outs during the preceding three months, number and qualifications of staff and any kind of malaria diagnosis and treatment training received by them. In the interview they were asked about availability of a microscope or RDTs for malaria, with confirmation of their responses by observation. The availability of trained laboratory technicians, however, was based on interviews with the person in charge of the health facility. Record analysis at various levels was also carried out to check the authenticity of the information.

### Data management and analysis

Data collected through semi-structured interviews and health facility assessments were entered by the field investigators into specially designed data management software. Data was entered on the same day as data collection as far as possible, and always before the study team left each district. Entered data were then exported to XML format and imported to MS Access 2007 (Microsoft Inc, Redmond, WA, USA). Data files were compared for errors by cross-checking with the original questionnaires. Data analysis was performed using SPSS 20.0.0 (SPSS Inc 1989-2007).

Descriptive analysis was conducted at health facility and health worker (ANM and ASHA) levels. As per state policy and from the epidemiological malaria data, the selected blocks in Categories-I and II should have had the same strategy for policy implementation. Analyses were therefore carried out considering Categories-I and II districts together as moderately to highly endemic districts, with a separate analysis for the non-endemic, Category-III district.

A series of analyses were conducted to assess the readiness of health workers to offer malaria diagnosis and treatment services. First, ANMs and ASHAs were considered to have the necessary knowledge to diagnose malaria if they knew all of the following: (1) to check the expiry date of the RDT; (2) which end of the RDT to dip in the buffer solution; and, (3) the correct interpretation of the RDT result, assessed using photographs of positive and negative tests. Amongst those with the necessary knowledge, readiness to perform diagnostic testing was determined by the availability of RDTs. Secondly, ANMs and ASHAs were considered to know how to treat malaria appropriately if they knew the drug of choice for treatment of malaria in children as well as adults correctly, in accordance with the recommended guidelines for malaria treatment. ANMs and ASHA with the necessary knowledge were then considered ready to treat malaria if they had any ACT available. Considering the high risk status of pregnant women and the possible foeto-toxic effect of anti-malarials, the State NVBDCP office has advised ANMs and ASHSs not to treat malaria positive pregnant mother and refer them to a nearest higher facility for treatment and close follow-up. Thus none of the ANM or ASHA are treating pregnant women.

Chi-square tests were used to assess differences in proportions, and logistic regression was used to determine associations between knowledge and readiness for diagnosis and treatment by considering factors such as age of the health worker, educational status, type of training received, time since training, availability of drugs for treatment, and the years of job experience. Differences were considered significant at a p-value of less than 0.05.

Ethical approval for the study was obtained from Institutional Ethics Committee of Public Health Foundation of India, New Delhi, and necessary permission was also obtained from the Government of Odisha. Written informed consent was obtained from all ANMs and ASHAs following an explanation of the study aims and procedures. An informed verbal consent was obtained from key stakeholders prior to interviewing them.

## Results

### Malaria in the selected blocks

**Figure 3:**
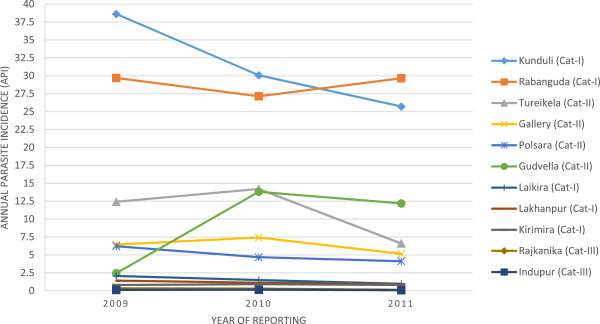
Annual parasite incidences in the sampled blocks (2009-2011).

Figure 3 shows that the API did not change substantially in most of the blocks between 2009 and 2011. The API crossed threshold values from 2009 to 2011 for different strategies in three blocks, one block in Category–I and two in Category-II districts.

### Health workers

A total of 29 health facilities including 11 (38%) CHCs and 18 (62%) PHCs were assessed. Of the 220 ANMs interviewed, 100 (45.4%), 73 (33.8%) and 47 (21.3%) came from districts in Categories-I, II and III, respectively. Similarly, of the 235 ASHAs interviewed, 42.5% (N = 100), 36.1% (N = 85) and 21.2% (N = 50) came from districts in Category-I, II and III, respectively.

The average age (range) of ANMs was 38.5 (22-59) years, compared with 33.8 (21-52) for ASHAs. While there was no significant difference in the age structure of ANMs in the three categories of districts, the ASHAs in the Category-III district were significantly older than those in Category-I and II (37.6 years *versus* 32.5 and 33.2 years, respectively, P < 0.001). The mean length (±SD) of schooling for ANMs and ASHAs was 11.4 (± 1.9) years and 8.2 (± 2.3) years respectively.

### Health worker readiness for malaria diagnosis

Table 1 shows that, of the 235 ASHAs interviewed, 89% (n = 209) were trained in malaria diagnosis and treatment. A significantly higher proportion of ASHAs were trained in malaria-endemic districts (Categories-I and II) than in the non-endemic district (94 *versus* 68%, P < 0.001). In contrast, the trained ANM proportion was as low as 32% in Category-III district to 49 and 67% in Categories-I and II districts. Nevertheless, a high proportion of ANMs (80%) and AHSAs (77%) had the necessary level of knowledge to be able to use RDTs for malaria diagnosis. The ANMs and ASHAs in endemic districts were more knowledgeable than their counterparts in non-endemic districts (P < 0.001). Over a third of ANMs and almost a half of ASHAs in moderately to highly endemic districts had no RDT kits. The RDTs that were available could diagnose only *P. falciparum* malaria, requiring all ANMs and ASHAs to collect blood slides from RDT-negative febrile patients and send them to CHCs for diagnosis of *P. vivax* infections.

**Table 1:** Training and knowledge of health workers in diagnosing fever cases

	**ANMs**			**ASHAs**		
	**Total**	**Category-I and II**	**Category-III**	**Total**	**Category-I and II**	**Category-III**
	**N = 220**	**N = 173**	**N = 47**	**N = 235**	**N = 185**	**N = 50**
	**Percentage (95% CI)**	**Percentage (95% CI)**	**Percentage (95% CI)**	**Percentage (95% CI)**	**Percentage (95% CI)**	**Percentage (95% CI)**
Ever attended ACT malaria case management training including RDT use	45.4 (38.9-52.0)	57.8 (50.3-65.0)	31.9 (19.8-46.1)	88.9 (84.4-92.4)	94.5 (90.5-97.2)	68 (54.2-79.7)
On-the-job training related to blood collection and slide preparation for malaria diagnosis	52.2 (45.6-58.8)	66.4 (59.1-73.2)	100 (93.8-100.0)	95.7 (92.5-97.8)	95.1 (91.2-97.6)	98 (90.5-99.3)
Access to malaria case management guidelines	61.8 (52.2-68.0)	65.8 (58.5-72.6)	46.8 (32.9-61.0)	71.0 (65.0-76.5)	81.0 (74.9-86.2)	34 (21.9-47.8)
**Knowledge about malaria diagnosis and use of RDT**						
1. Know that malaria is diagnosed with either slide microscopy or RDT	100 (98.6-100.0)	100 (98.2-100.0)	100 (93.8-100.0)	100 (98.7-100.0)	100 (98.3-100.0)	100 (94.1-100.0)
2. Know how to check that RDT is okay for use	87.2 (82.3-91.2)	98.2 (95.3-99.5)	46.8 (32.9-61.0)	87.2 (82.5-91.0)	93.5 (89.2-96.4)	64.0 (50.0-76.3)
3. Know which side of RDT strip to dip into the buffer solution	81.8 (76.3-86.5)	90.7 (85.7-94.4)	48.9 (34.9-63.0)	79.5 (74.0-84.3)	90.2 (85.3-93.9)	40.0 (27.1-53.9)
4. Able to interpret RDT result correctly	86.8 (81.8-90.8)	98.2 (95.3-99.5)	44.6 (31.0-59.0)	86.8 (82.0-90.7)	94.0 (89.9-96.8)	60.0 (46.0-72.8)
Acceptable level of knowledge for diagnosing malaria using RDT	80.0 (74.3-84.8)	90.2 (85.0-93.9)	42.6 (29.0-56.9)	77.4 (71.7-82.4)	88.1 (82.8-92.2)	38.0 (25.4-51.9)
Ready to diagnose fever cases using RDTs*	45.0 (38.5-51.6)	57.2 (49.7-64.4)	0	39.5 (33.4-45.9)	50.2 (43.0-57.4)	0

* A combination of acceptable knowledge and availability of RDT or microscopy.

All laboratory technicians in CHCs were trained in malaria microscopy and the use of RDT kits, compared with only 30% of the 36 medical officers. Nine of the 11 CHCs had both microscopy and RDTs available for malaria diagnosis. In contrast, none of the two CHCs in the non-endemic district had RDTs on the day of survey and only one had microscopy available, due to non-availability of laboratory technicians. Sixteen out of 18 PHCs did not have microscopy available due to the lack of a functioning laboratory or technician, as there is no sanctioned post of laboratory technician at PHC level in Odisha. In addition, two-thirds of PHCs did not have RDTs available on the day of survey.

### System readiness to treat malaria

It was observed that all of the interviewed ANMs and ASHAs in non-endemic (Category-III) districts referred malaria-positive patients to health facilities for treatment. Of more concern is that two-thirds (64%) of ASHAs and almost half (43%) of ANMs from endemic districts referred *P. falciparum-*positive malaria cases to health facility (Table 2). Only 35% of ASHAs and two-thirds of ANMs were treating uncomplicated *P. falciparum-*positive, non-pregnant adults as per the policy. The proportions of ASHAs and ANMs who practiced radical treatment for *P. vivax* were also low as 24% for ASHAs and 47% for ANMs. Multivariate analysis did not identify age of the health worker, their educational status, type of training they received, duration since training, availability of drugs for treatment or years of job experience to be significantly associated with knowledge and readiness for diagnosis and treatment at ANM and ASHA level.

**Table 2:** Current auxiliary nurse midwives and accredited social health activists treatment strategy for uncomplicated malaria

	**ANMs**			**ASHAs**		
	**Total**	**Category-I**	**Category-II**	**Total**	**Category-I**	**Category-II**
	**N = 220**	**N = 100**	**N = 73**	**N = 235**	**N = 100**	**N = 85**
	**Percentage (95% CI)**	**Percentage (95% CI)**	**Percentage (95% CI)**	**Percentage (95% CI)**	**Percentage (95% CI)**	**Percentage (95% CI)**
**Falciparum malaria**						
Treatment of children for falciparum infection as per policy	0	0	0	0	0	0
Treatment of non-pregnant adult for falciparum infection as per the policy	47.7 (41.1-54.3)	72.0 (62.6-80.1)	45.2 (34.0-56.7)	27.6 (22.2-33.6)	48.0 (38.3-57.7)	20.0 (12.5-29.5)
Refer falciparum-positive children and non-pregnant adults patient for treatment to health facility	55.0 (48.3-61.4)	38.0 (28.8-47.7)	50.6 (39.3-62.0)	71.4 (65.4-76.9)	60.0 (50.1-69.2)	68.2 (57.7-77.4)
**Vivax malaria**						
Treatment of vivax infection in children as per policy	02.7 (01.1-05.5)	02.0 (0.33-6.45)	05.5 (01.7-12.6)	0.42 (0.02-2.08)	0	1.1 (0.05-5.6)
Treatment of vivax infection in non-pregnant adults as per policy	47.2 (40.7-53.8)	66.0 (56.3-74.7)	52.0 (40.6-63.3)	24.2 (19.1-30.0)	34.0 (25.2-43.6)	27.0 (18.4-37.2)
Refer vivax malaria-positive children and non-pregnant adults patient for treatment to health facility	60.9 (54.3-67.2)	48.0 (38.3-57.7)	53.4 (41.9-64.6)	75.7 (69.9-80.9)	65.0 (55.2-73.8)	74.1 (64.0-82.5)

More than two-thirds (71%) of ASHAs and 61.8% of ANMs had access to the treatment guidelines. Access to guidelines was significantly higher in ANMs and ASHAs of Category-I district compared to Category-II and Category-III (70 *versus* 60 and 47%, P = 0.025 for ANMs; and 93 *versus* 67 and 34%, P < 0.001 respectively for ASHAs).

Almost two thirds of ASHAs and over one-third of ANMs did not have any ACT in Categories I and II districts. Furthermore, only one-third (34%) of these ANMs, and less than a fifth (15%) of these ASHAs, had all five age packs of ACT. In the same districts, 22% of ANMs and 39% of ASHAs had no CQ tablets, and 40% of ANMs and 80% of ASHAs had no PQ tablets for radical cure of malaria. None of the ANMs and ASHAs in the Category-III district had PQ tablets. Additional file 1: Table S1 shows the availability of anti-malarials and diagnostics on the day of the survey and their stock-outs in preceding three months in more detail.

Two-thirds of ASHAs and 43% of ANMs from endemic districts referred *P. falciparum-*positive malaria cases to health facilities. The proportions of ASHAs and ANMs who practiced radical treatment for *P. vivax* were also low at 24 and 47%, respectively (Table 2).

Two-thirds (19 out of 29) of health facilities had at least some ACT blister packs, although only eight (73%) CHCs and six (33.3%) PHCs had all strengths of ACT available. The duration of the stock-outs for different strengths of ACT ranged from one month for the five to eight years and nine to 14 years age packs to ten months for the infant and one to four years packs, in different facilities. Only four out of 29 health facilities had artemisinin injections for treatment of severe complicated malaria. The availability of age-specific ACT packs at subcentre level in Category-I districts varied from 54% for the infants pack to 76% for the adult pack, compared with 18% (for the infants pack) to 36% (for the adult tablet pack) in Category-II districts. Neither the health facilities nor the health workers in the Category-III districts had any ACT tablets.

### Health system readiness to deliver the malaria diagnosis and treatment policy

**Figure 4:**
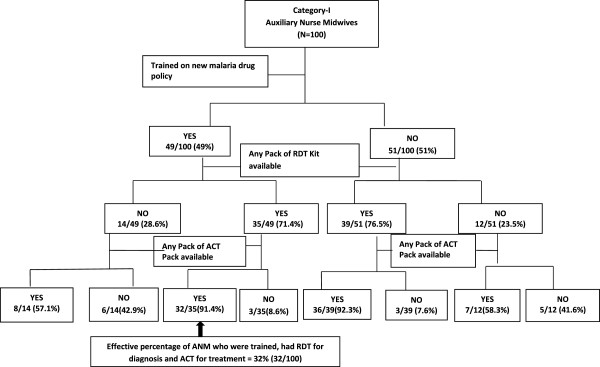
Readiness of auxiliary nurse midwives to implement new malaria drug policy.

An appropriately knowledgeable individual who had a supply of RDTs was considered ready to offer malaria diagnosis: only half of the ANMs and ASHAs fell into this category despite ~80% of them having the required knowledge. Extending this to treatment, the percentage of ANMs who were trained, had RDTs for diagnosis and ACT for treatment of *P. falciparum* cases was 32, 17 and 0% in Categories-I, II and III districts, respectively (Figure 4). The corresponding proportions of ASHAs were 48, 10 and 0%.

### Supervision and reporting of cases

Of the 220 ANMs and 235 ASHAs interviewed, 62% of ANMs and 37% of ASHA in malaria-endemic districts had submitted their monthly report form in the prescribed format and on time (Table 3). None of the ASHAs and only 12% of the ANMs in the non-malaria endemic district had submitted their monthly report on time. There was no significant difference in the proportion of ANMs in moderately to highly endemic districts as compared to the non-endemic district who had supervision in which malaria case management was a discussion topic (49.7 *versus* 36.1%, P = 0.09).

**Table 3:** Submission of monthly report by health workers and their supervision in preceding three months

	**Categories-I and II**	**Category-III**	**Total**
	**ANM (N = 173)**	**ANM (N = 47)**	**ANM (N = 220)**
	**ASHA (N = 185)**	**ASHA (N = 50)**	**ASHA (N = 235)**
	**Per cent (95% CI)**	**Per cent (95% CI)**	**Per cent (95% CI)**
**Submitted last due report in required format**			
**ANM**	71.6 (64.6-78.0)	51.1 (36.9-65.0)	67.2 (60.6-73.2)
**ASHA**	47.0 (39.9-54.2)	0	37.0 (31.0-43.3)
**On time report submission status**			
**ANM**	62.4 (55.0-69.4)	12.7 (5.3-24.6)	65.8 (58.5-72.6)
**ASHA**	36.7 (30.0-43.8)	0	36.7 (30.0-43.8)
**Any supervision visit in preceding three months**			
**ANM**	60.1 (52.6-67.2)	74.4 (60.6-85.3)	63.1 (56.6-69.3)
**ASHA**	42.7 (35.7-49.9)	22.0 (12.1-35.0)	38.2 (32.2-44.6)
**Any supervision visit in preceding three months which included discussion about malaria case management and control**			
**ANM**	49.7 (42.3-57.1)	36.1 (23.4-50.5)	46.8 (40.2-53.4)
**ASHA**	37.2 (30.5-44.4)	08.0 (02.5-18.1)	31.0 (25.3-37.2)

## Discussion

This study assessed the health system’s readiness to implement a malaria drug and diagnosis policy in Odisha State of India. Readiness was assessed in terms of the availability of trained human resources, drugs and diagnostics. This assessment revealed a number of important findings which may be considered in further strengthening the management of fever patients in general, and malaria in particular, in Odisha. The most striking finding was that, despite a high level of knowledge about how best to diagnose and treat malaria, the ability of the peripheral health workers to optimize fever management and malaria diagnosis was compromised by a failure of the supply chain. Whereas 80% of field-level workers (ASHAs and ANMs) knew how to diagnose malaria, only 40% were provided with the tools to put this knowledge into effective practice.

The API, which is a measure of malaria endemicity, did not change remarkably in most of the sampled blocks in the three years preceding this study. In the few blocks that had changes in API, these might have been due to variation in surveillance activities. With increased active surveillance the annual blood examination rates (ABER) increases and the chances of having a higher API (higher positive cases) also increases in endemic regions. One block in Category-II crossed the policy threshold for deployment of ACT and RDTs at the ASHA level for API of the three years. There is a need to consider how frequently the strategy of policy implementation should be reviewed.

The critical prerequisite for effective implementation of any treatment policy is to develop a trained workforce to implement it and make the recommended, non-expired medicines universally available. This study revealed that the majority of ASHAs in all three categories were trained in malaria case management, while less than half of the ANMs were trained in the new malaria treatment policy. This may reflect that ANMs are considered to be trained on malaria diagnosis and treatment during their training course before joining the service. However, it was seen that the majority of ANMs were aware of the change in malaria treatment policy, despite a lack of formal training, probably through routine sensitization during their monthly review meetings at the CHCs. This is also where they had learnt to use RDTs.

Early diagnosis and complete treatment is a key component of malaria control. The recommendation in India is that every *P. falciparum* case should be treated with ACT and this be accompanied by single dose PQ on day two, except for pregnant women and infants. None of the ANMs and ASHAs was treating children according to the policy, and that a high proportion of ANMs and ASHAs were referring malaria-positive patients to health facilities for treatment. This is suboptimal as it delays treatment of this life-threatening disease [15] and unnecessarily burdens the higher referral facilities. There is also a considerable risk that the referred patient does not contact the health facility.

A key element of successful policy implementation, and in turn effectiveness, is that the recommended drugs should be available at the point of care [7,8]. The key strategy in the 2010 malaria drug policy is to make RDTs and anti-malarials available to ANMs and ASHAs for early diagnosis and complete treatment of each malaria case. The finding that half (48%) of the facilities did not stock all strengths of ACT is worrisome, especially when only one-third of ANMs and less than a fifth of ASHAs had all the age-specific packs available. To ensure a reliable supply of important drugs, such as ACT, the NVBDCP has set norms for the deployment of a reserve to be kept at each level [16]. As per the norms, every ASHA should have two ACT treatment packs for each age group, and the subcentre should keep three packs of each paediatric dosage and six packs for adults. However, less than a third of ANMs and ASHAs had at least one pack from each age group of ACT blister pack with them. A high proportion of subcentres and ASHAs had experienced stock-outs of ACT, RDTs and CQ in the preceding three months indicating the irregularity in the supply chain. The magnitude of anti-malarial stock-outs found in this study is not peculiar to Odisha; similar findings have been documented in various studies from many malaria-endemic African countries [17-20]. Stock-outs are not only related to ACT; 80% of ASHAs and 40% of ANMs in malaria-endemic regions had no PQ tablets on the day of survey and a significant proportion of ANMs also reported stock-outs. A discussion with the primary stakeholders has shown that the stock-outs were mainly due to the delay in tendering and procurement processes. Currently the procurement of drugs and diagnostics (RDTs) are mainly carried out at national level by Central NVBDCP office and then supplied to different states. Sometimes there are interrupted supplies which lead to shortage of drugs at the peripheral level. Future studies evaluating qualitative and quantitative characteristics of ACT and other drug supply chains are required to better understand causes of stock-outs and learn how best to streamline the process.

The relatively high level of knowledge about how to diagnose at grass-roots level was undermined by the poor availability of RDTs. More than 80% of ANMs and ASHAs in endemic districts had an acceptable level of knowledge on malaria diagnosis, but there was a 30 to 50% reduction in their readiness level as a result of the supply chain failure. Malaria diagnostic services were limited to CHCs and above. Two-thirds of PHCs were without RDTs, which is a serious flaw considering this level of care is supposed to provide laboratory services as per the Indian Public Health Standards (IPHS). The absence of malaria microscopy at the PHC level and inability on the part of ASHAs and ANMs to send blood slides for parasitological diagnosis as soon as the slide is collected is creating a burden on existing laboratories at CHCs. It should be possible to increase rapidly the coverage of health facilities with malaria diagnostics by focusing on large-scale procurement and supply of RDTs together with quality assurance activities to those facilities with malaria microscopy.

The existing national guideline for forecasting RDT need is based on the assumption that 40% of all blood slides collected for malaria diagnosis are from remote, hard-to-reach areas [16]. Like most other malaria-endemic countries, malaria diagnostic services are part of the general health care system. Therefore, strengthening microscopic facilities for diagnosis of malaria is an opportunity for improving the existing health care system. The use of RDTs or microscopy should therefore not be intended as a stand-alone activity, but fully integrated as part of an overall effort to strengthen laboratory services.

Lack of microscopy facilities at PHCs creates an undue delay in reporting the results of slide microscopy. This is required primarily to identify non-falciparum species, an important consideration as 10-25% of malaria infections are due to *P. vivax* in Odisha [21-23]. Supply of bivalent RDTs, of adequate sensitivity to diagnose both *P. falciparum* and *P. vivax* infection at the grass-root level could solve the problem to a large extent and will also reduce the existing burden on the microscopy centres.

Provision of ACT and diagnostics requires a gamut of activities and strong health system support for health workers to implement, reinforce and maintain effective malaria case management practices. This study findings revealed gaps in the coverage of health workers with malaria-related health systems support activities; 30 to 40% of ANMs in malaria-endemic districts and 30% of ASHAs had not attended ACT-based case-management training and instruction in the use of RDTs; one-third of ANMs and ASHAs had no access to treatment protocols; one-third of ANMs and 60% of ASHAs had not received any supervisory visits and more importantly only 47% of ANMs and one-third of ASHAs received a visit that included any activity related to malaria case management. An opportunity to increase health workers exposure to these activities lies in ensuring the availability of RDTs, which should be accompanied by broader and comprehensive malaria case management and in-service training, dissemination of job aids, post-training follow-up, structured supervisory visits and performance monitoring.

Some caveats need to be considered in the interpretation of the findings of this study. First, the survey was undertaken once, during February to July 2012, and as such the results represent a single snapshot of the status of service provision in Odisha. Seasonal variation in system readiness may have been missed. However, the deficits identified warrant attention and systems need strengthening to optimize system performance at all times of year. Second, the study did not include malaria cases for interview, which could have provided further insights into the reality of diagnosis and treatment practices in Odisha State. Finally, for logistic and financial reasons, the study was undertaken in only five districts and may not be fully representative of the entire State of Odisha. Steps were taken during stratification to ensure representativeness of the State, so the results should be generalizable to a reasonable degree.

## Conclusion

Two years after the introduction of the new malaria drug policy, there are several gaps in the health system, which together limit the effectiveness of malaria case management activities. Shortfalls in the availability of drugs and diagnostic testing can be attributed in part to the relatively recent policy change and the expected time lag in securing funds, subsequent procurement of RDTs and assuring their availability at the community level. The majority of ASHAs had been trained in malaria case management and had a relatively high level of knowledge, but their potential to perform was undermined by the poor availability of RDTs, ACT and PQ. In addition, the poor availability of drugs at community level resulted in a high rate of referral, which is a major hindrance in achieving early diagnosis and complete treatment of malaria. The programme should consider a web-based monitoring, as well as tracking, system for stock-out of drugs and logistics. In addition, a de-centralized mechanism for procurement and supply of commodities should also be considered. The findings of this study have been shared with the State NVBDCP office, and they have expressed interest in using these findings for further programme development and future planning.

## Competing interests

The authors have declared that they have no competing interests.

## Authors’ contributions

MAH conceived the study and was guided by fellowship supervisors LD and DS on the study design, data collection, analysis, interpretation of findings, and drafting of the manuscript. All authors read and approved the final manuscript.

## Supplementary Material

Additional file 1: Table S1 Availability of anti-malarials and diagnostics on the day of survey and their stock-outs in preceding three months. Click here for file

## References

[B1] WHO World malaria report 2012 2012 Geneva: World Health Organization

[B2] Dhingra N Jha P Sharma VP Cohen AA Jotkar RM Rodriguez PS Bassani DG Suraweera W Laxminarayan R Peto R Adult and child malaria mortality in India: a nationally representative mortality survey Lancet 2010 376 1768 1774 10.1016/S0140-6736(10)60831-8 20970179 PMC3021416

[B3] WHO World malaria report 2009 2009 Geneva: World Health Organization

[B4] Dash AP Valecha N Anvikar AR Kumar A Malaria in India: challenges and opportunities J Biosci 2008 33 583 592 10.1007/s12038-008-0076-x 19208983

[B5] Sharma RS Sharma GK Dhillon GPS Epidemiology and control of malaria in India 1996 New Delhi: Directorate of National Malaria Eradication Programme

[B6] National Drug Policy on Malaria 2010 http://nvbdcp.gov.in/Doc/drug-policy-2010.pdf

[B7] Amin AA Zurovac D Kangwana BB Greenfield J Otieno DN Akhwale WS Snow RW The challenges of changing national malaria drug policy to artemisinin-based combinations in Kenya Malar J 2007 6 72 10.1186/1475-2875-6-72 17535417 PMC1892027

[B8] Williams HA Durrheim D Shretta R The process of changing national malaria treatment policy: lessons from country-level studies Health Policy Plan 2004 19 356 370 10.1093/heapol/czh051 15459161

[B9] Whitty CJ Chandler C Ansah E Leslie T Staedke SG Deployment of ACT antimalarials for treatment of malaria: challenges and opportunities Malar J 2008 7 S7 10.1186/1475-2875-7-S1-S7 19091041 PMC2604871

[B10] Zurovac D Ndhlovu M Sipilanyambe N Chanda P Hamer DH Simon JL Snow RW Paediatric malaria case-management with artemether-lumefantrine in Zambia: a repeat cross-sectional study Malar J 2007 6 31 10.1186/1475-2875-6-31 17367518 PMC1832199

[B11] Zurovac D Tibenderana JK Nankabirwa J Ssekitooleko J Njogu JN Rwakimari JB Meek S Talisuna A Snow RW Malaria case-management under artemether-lumefantrine treatment policy in Uganda Malar J 2008 7 181 10.1186/1475-2875-7-181 18803833 PMC2556699

[B12] Zurovac D Njogu J Akhwale W Hamer DH Snow RW Translation of artemether-lumefantrine treatment policy into paediatric clinical practice: an early experience from Kenya Trop Med Int Health 2008 13 99 107 10.1111/j.1365-3156.2007.01980.x 18291008 PMC2592474

[B13] Census of India Provisional Population Totals Paper 1 of 2011: Odisha. Office of the Registrar General and Cencus Comissioner, India. Government of Indiahttp://www.censusindia.gov.in/2011-prov-results/prov_data_products_odisha.html

[B14] Malaria Situation in Indiahttp://nvbdcp.gov.in/malaria-new.html

[B15] Das A Gupta RD Friedman J Pradhan MM Mohapatra CC Sandhibigraha D Community perceptions on malaria and care-seeking practices in endemic Indian settings: policy implications for the malaria control programme Malar J 2013 12 39 10.1186/1475-2875-12-39 23360508 PMC3570348

[B16] Directorate of National Vector Borne Disease Control Programme Operational Manual for Implementation of Malaria Programme 2009 New Delhi: Directorate General of Health Services, Government of India http://nvbdcp.gov.in/Doc/Malaria-Operational-Manual-2009.pdf

[B17] Njogu J Akhwale W Hamer DH Zurovac D Health facility and health worker readiness to deliver new national treatment policy for malaria in Kenya East Afr Med J 2008 85 213 221 18814531 10.4314/eamj.v85i5.9615 PMC2669166

[B18] Abdelgader TM Ibrahim AM Elmardi KA Githinji S Zurovac D Snow RW Noor AM Progress towards implementation of ACT malaria case-management in public health facilities in the Republic of Sudan: a cluster-sample survey BMC Public Health 2012 12 11 10.1186/1471-2458-12-11 22221821 PMC3268707

[B19] Nyandigisi A Memusi D Mbithi A Ang’wa N Shieshia M Muturi A Sudoi R Githinji S Juma E Zurovac D Malaria case-management following change of policy to universal parasitological diagnosis and targeted artemisinin-based combination therapy in Kenya PLoS One 2011 6 e24781 10.1371/journal.pone.0024781 21935464 PMC3173476

[B20] Kangwana BB Njogu J Wasunna B Kedenge SV Memusi DN Goodman CA Zurovac D Snow RW Malaria drug shortages in Kenya: a major failure to provide access to effective treatment Am J Trop Med Hyg 2009 80 737 738 19407116 PMC2679204

[B21] Sharma SK Chattopadhyay R Chakrabarti K Pati SS Srivastava VK Tyagi PK Mahanty S Misra SK Adak T Das BS Chitnis CE Epidemiology of malaria transmission and development of natural immunity in a malaria-endemic village, San Dulakudar, in Orissa state, India Am J Trop Med Hyg 2004 71 457 465 15516643

[B22] Sharma SK Tyagi PK Padhan K Upadhyay AK Haque MA Nanda N Joshi H Biswas S Adak T Das BS Chauhan VS Chitnis CE Subbarao SK Epidemiology of malaria transmission in forest and plain ecotype villages in Sundargarh District, Orissa, India Trans R Soc Trop Med Hyg 2006 100 917 925 10.1016/j.trstmh.2006.01.007 16697022

[B23] Sahu SS Gunasekaran K Vanamail P Jambulingam P Persistent foci of falciparum malaria among tribes over two decades in Koraput district of Odisha State, India Malar J 2013 12 72 10.1186/1475-2875-12-72 23433186 PMC3598688

