# Supplementary material for: Public health system readiness to treat malaria in Odisha State of India
Source: Malar J. 2013 Oct 2;12:351. doi: 10.1186/1475-2875-12-351 (PMC3850888; doi:10.1186/1475-2875-12-351)
Supplement: Additional file 1: Table S1 — Availability of anti-malarials and diagnostics on the day of survey and their stock-outs in preceding three months. [file 1475-2875-12-351-S1.docx]

**Additional file 1: Table S1 Availability of anti-malarials and diagnostics on the day of survey and their stock-outs in preceding three months**

|  | **ANMs (N=220)** | | **ASHAs (N=235)** | |
| --- | --- | --- | --- | --- |
|  | **Categories-I and II**  **N=173**  **Per cent (95% CI)** | **Category-III**  **N=47**  **Per cent(95% CI)** | **Categories-I and II**  **N=185**  **Per cent (95% CI)** | **Category-III**  **N=50**  **Per cent (95% CI)** |
| **Availability of any amount of anti-malarials on the day of survey** |  | | | |
| Any tablet pack of ACT | 64.2(56.8-71.0) | - | 38.3(31.5-45.5) | - |
| ACT infant pack | 30.6(30.5-44.9) | - | 15.1(10.5-20.8) | - |
| ACT pack for 1-4 years children | 49.7(42.3-57.1) | - | 25.9(20.0-32.6) | - |
| ACT pack for 5-8 years children | 50.8(43.4-58.2) | - | 25.4(19.5-32.0) | - |
| ACT pack for 9-14 years children | 51.4(44.0-58.8) | - | 26.4(20.5-33.2) | - |
| ACT adult pack | 59.0(51.5-66.1) | - | 38.3(31.5-45.5) | - |
| At least one pack each from all strength | 34.1(27.3-41.4) | - | 15.1(10.5-20.8) | - |
| Chloroquine tablets | 78.0(71.4-83.7) | 63.8(49.4-76.5) | 61.0(53.9-67.9) | 36.0 (23.6-49.9) |
| Primaquine tablets (any strength) | 60.1(52.6-67.2) | - | 20.5(15.1-26.8) | - |
| RDT kits for malaria diagnosis | 62.4(55.0-69.4) | - | 55.6(48.4-62.7) | - |
| **Stock-out of anti-malarials drugs and RDTs in preceding three months from the day of survey** |  |  |  |  |
| Any tablet pack of ACT | 64.1(56.8-71.0) | 100(93.8-100.0) | 81.0(74.9-86.2) | 100.0 (94.1-100) |
| Chloroquine tablets | 36.4(29.5-43.7) | 47.5(32.4-62.8) | 58.9(51.7-65.8) | 70.0 (56.3-81.4) |
| RDTs | 71.6(64.6-78.0) | 100(93.8-100.0) | 77.2(70.8-82.9) | 100.0 (94.1-100) |
